# The Moderating Effect of Caregiver Engagement in Transitional Care Intervention Outcomes: A Meta-Analysis

**DOI:** 10.1093/geroni/igab046.1382

**Published:** 2021-12-17

**Authors:** Kristin Levoy, Eleanor Rivera, Molly McHugh, Alexandra Hanlon, Karen Hirschman, Mary Naylor

**Affiliations:** 1 University of Indiana School of Nursing (IUPUI Campus), Indianapolis, Indiana, United States; 2 University of Illinois Chicago, Chicago, Illinois, United States; 3 University of Pennsylvania, Philadelphia, Pennsylvania, United States; 4 Virginia Tech, Roanoke, Virginia, United States

## Abstract

As chronically ill adults age, increased fluctuations in health status result in frequent care transitions. Caregiver engagement is often a core component of evidence-based transitional care interventions, yet little is known about the relative contribution of this element to observed outcomes. This meta-analysis aimed to synthesize evidence of caregiver engagement in randomized control trials (RCT’s) of transitional care interventions, estimate the overall intervention effects on all-cause hospital readmissions, and test caregiver engagement as a moderator of interventions’ effects. Relative risk was the effect size, and the overall effect was estimated using inverse variance weighting. Fifty-four studies met criteria, representing 31,399 participants and 65 effect sizes. The weighted sample mean age was 64 years. The majority (64%) of interventions targeted participants with specific diagnoses, such as heart disease, but more than half (54%) lacked caregiver engagement components. Among all reviewed studies of transitional care interventions, the overall effect on all-cause readmissions at 1 month was non-significant (p=.123, k=28). However, intervention effects at 2 or more months were significant (RR=0.89, 95% CI: 0.82, 0.97, p=.007, k=26), indicating a 12% reduction in the relative risk of all-cause readmissions among intervention participants compared to controls. Caregiver engagement was found to moderate intervention effects (p=.05). Specifically, interventions that included caregiver engagement produced more robust effects (RR=0.83, 95% CI: 0.75, 0.92, p=.001), than those without such engagement (RR=0.97, 95% CI: 0.87, 1.08, p=.550). Findings suggest that transitional care interventions need to more explicitly engage caregivers as active partners in order to optimize patient outcomes.

